# Using 360-degree video for teaching emergency medicine during and beyond the COVID-19 pandemic

**DOI:** 10.1080/07853890.2021.1970219

**Published:** 2021-10-06

**Authors:** Alina Petrica, Diana Lungeanu, Alexandru Ciuta, Adina M. Marza, Mihai-Octavian Botea, Ovidiu A. Mederle

**Affiliations:** a “Victor Babes” University of Medicine and Pharmacy, Timisoara, Romania; b “Pius Brinzeu” Emergency Clinical County Hospital, Timisoara, Romania; c Center for Modeling Biological Systems and Data Analysis, Department of Functional Sciences, “Victor Babes” University of Medicine and Pharmacy, Timisoara, Romania; d Multidisciplinary Center for Research, Evaluation, Diagnosis, and Therapies in Oral Medicine, Department of Surgery, Faculty of Medicine, “Victor Babes” University of Medicine and Pharmacy, Timisoara, Romania; e Emergency Clinical Municipal Hospital, Timisoara, Romania; f Department of Surgery, Faculty of Medicine and Pharmacy, University of Oradea, Oradea, Romania

**Keywords:** Medical education, instructional films and video, emergency medicine training, 360-degree videos, video scenarios

## Abstract

**Objective:**

During the COVID-19 pandemic, emergency medicine (EM) teachers had to employ innovative methods to ensure the continuity of the education process. The purpose of this study was to explore the adequacy of the 360-degree video (video 360) technology in EM education in the context of: (a) students' attitudes towards the video 360; (b) students' academic performance in their required examination at the end of the EM course compared to the assessment results of students from the previous academic year.

**Methods:**

A mixed-method research project enrolled the fourth-year medical students who attended the required EM course during the first semester of the academic year 2020–2021 when all activities with undergraduate students went online and teaching scenarios recorded in the video 360 format were employed. Data collection was two-fold: (a) anonymous questionnaires, complemented with basic YouTube analytics; (b) multiple-choice questionnaires (MCQ) and oral examination, contrasting the results with those in 2019–2020. Data analysis used descriptive statistics and non-parametric methods.

**Results:**

Seventy-nine students (53 females and 26 males) participated in the project and all completed the EM course. Students' interest in and their acceptance of the video 360 technology were high (total scoring in the upper 20% of the respective scales), with consistently good performance in two parallel, independent, interview-based oral/practical evaluations (Spearman correlation coefficient *R* = 0.665, *p* < .001). The majority scored over 90% in the summative MCQ, with higher values compared to their colleagues’ during the previous academic year (with on-site teaching): scoring percentages with mean ± standard deviation of 92.52 ± 4.57 and 76.67 ± 18.77, respectively.

**Conclusion:**

Our project showed that the video 360 scenarios were effective in teaching EM. In the long term, employing this accessible and inexpensive educational approach would add value to on-site training by enriching the exposure to a specific ED environment.

**KEY MESSAGES**
- Medical students valued the 360-degree video scenarios as contributing substantially to their EM knowledge and preparedness.
- Examination results confirmed the 360-degree video scenarios as viable in EM teaching.
- The 360-degree video technology would be a sustainable solution for hybrid medical teaching in the long term.

## Graphical Abstract

**Figure:**
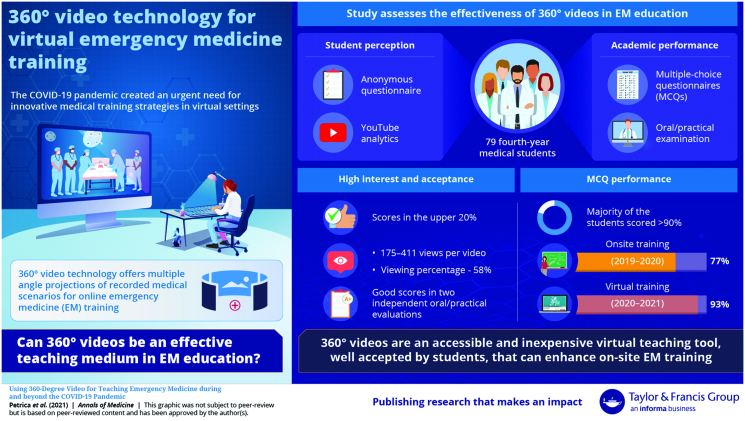

## Introduction

The COVID-19 pandemic forced educational institutions worldwide to shift their entire teaching process online, and in an extremely short time—a challenging and stressful situation for both teachers and students [1–6]. For emergency medicine (EM) educators, the challenge was even greater, as this specialty offers a truly unique educational experience with an endless stream of patients and diverse pathologies. EM education is stimulating in two directions: on the one hand, merging large volumes of medical knowledge; on the other hand, developing practical abilities like prioritisation and effective task-switching to manage patient overload or the general chaos in the department. Apart from learning to care for critically ill patients, students in the emergency department (ED) are also exposed to non-medical skills like time management, conflict resolution, teamwork, situational awareness, supervising and providing feedback, leadership, maintaining standards, assertiveness, and decision making. The ED is a rich learning environment, prone to spontaneous case-based teaching, with constant assaults on all the senses, and distractions unparalleled in the world of education [7,8].

Different technologies can be employed as educational tools in EM teaching and learning, like video games, computer simulations, mobile applications, virtual reality (VR), and augmented reality (AR). VR employs specialised software to create simulated environments that are completely separated from normal reality [9]. In contrast, AR uses superimposed layers of computer-generated enhancements to an existing reality to make the experience more meaningful and interactive [10]. The potential of VR and computer simulations for education has been explored in several studies [9], which proved the benefits of knowledge improvement, mastery of skills, and student satisfaction. VR is appropriate for training in high-acuity or rare events, such as disasters or mass casualty events, or rare clinical encounters and procedures.

Immersive videos or 360-degree videos (hereinafter video 360) are recorded using omnidirectional cameras and offer the possibility of virtual on-site experience by using VR devices, or just inexpensive flat screens of smartphones or laptops, with no dedicated software requirements. The recorded environment can be explored from different angles, repeatedly, and at one's own pace, which can be a valuable experience for EM trainees [11–15]. Compared to VR and AR, in the case of video 360, users cannot move within a scene or interact directly with objects in the environment. Compared to traditional video, the editing and post-production process of video 360 recordings is labour-intensive and complex, requiring powerful computers, with no actual standards to follow. The available literature on the use of video 360 in education mostly emphasises its advantages. However, Pirker and Dengel [16] identified the issue of small sample sizes and low statistical power in the majority of reported studies.

The medical curriculum includes a required EM course aimed at training all students in the basics of emergency care (from recognising and assessing a critical patient, making a differential diagnosis, and performing simple procedures, to managing life-threatening conditions). During the COVID-19 outbreak, all teaching for undergraduate students went online at our university, which confronted us with unexpected challenges regarding the necessary clinical exposure and practice. In this demanding context, employing recorded educational scenarios was an attainable approach; their effectiveness was a significant concern we decided to address.

We conducted a one-semester pilot educational project aimed at exploring the feasibility and effectiveness of employing recorded scenarios filmed using the video 360 technology in EM teaching routine. The purpose was two-fold: (a) to survey students' opinions and attitudes towards the use of the video 360 technology in teaching, including their interest in independently accessing the recorded scenarios; and (b) to measure the students' academic performance in their required examination at the end of the course and refer it to the previous academic year for comparison.

## Methods

### Design of research

This mixed-method educational project employed a two-arm research framework: (a) a cross-sectional survey-based investigation of students' perceptions and attitudes towards the use of the video 360 technology in medical education (in general) and EM (in particular); and (b) a descriptive, summative assessment of the intended outcomes of the course regarding students' understanding of the EM concepts; a comparison was made between the academic assessment results of two student cohorts for which on-site and online teaching had been used (in January 2020 and January 2021, respectively).

**Figure 1.**
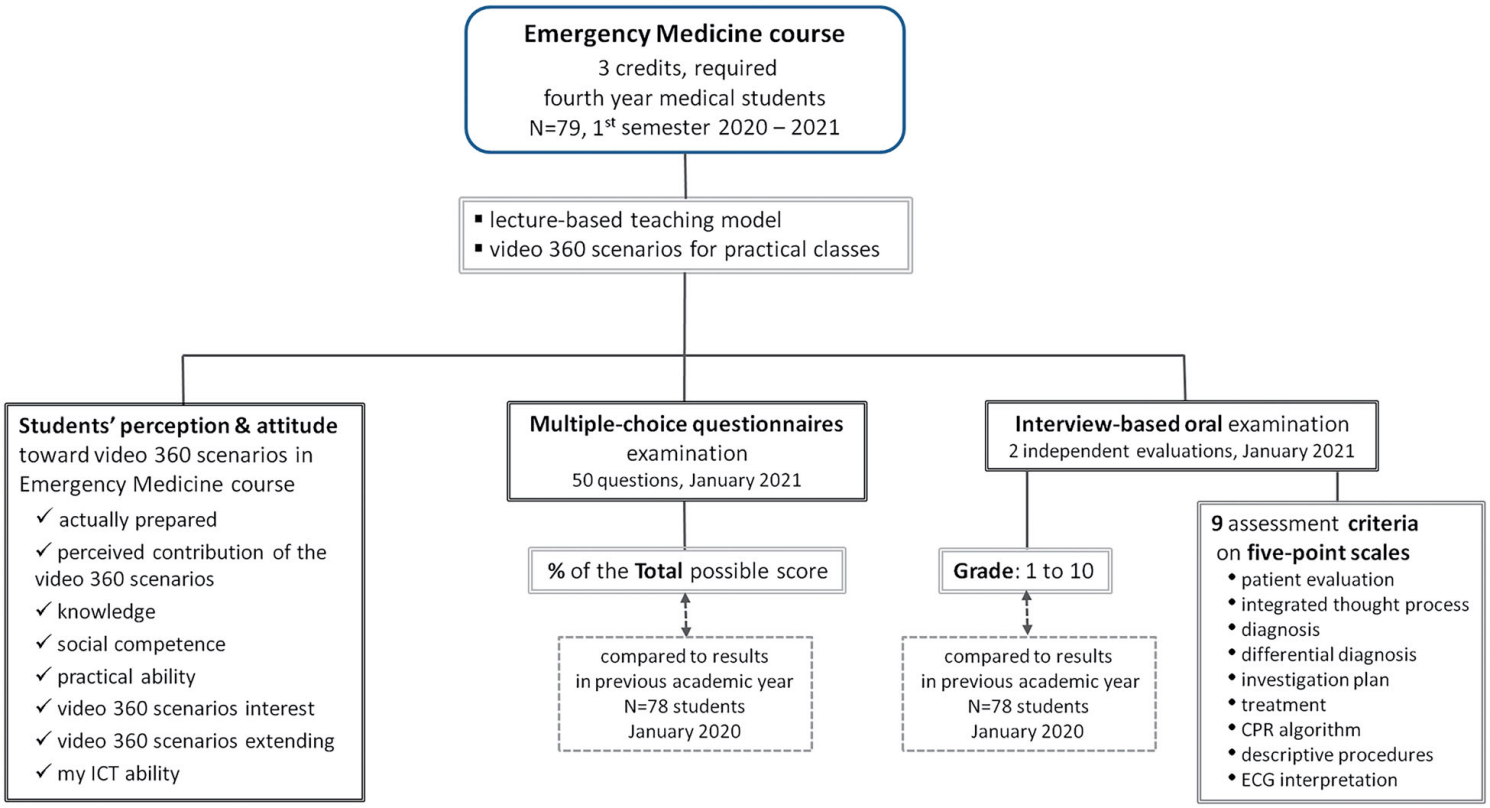
Study flow diagram. The three arms show the framework of data collection: (a) students' attitudes towards the video 360 technology, its appropriateness in EM, and its general effectiveness, by online anonymous questionnaires; (b1) students' assessment by multiple-choice questionnaires; (b2) students' assessment by interview-based oral examination. Abbreviations: CPR: Cardio-Pulmonary Resuscitation; ECG: electrocardiogram; ICT: Information and Communication Technology.

Figure 1 shows the study flow diagram for data collection in this project. Students' knowledge assessments using multiple-choice questionnaire (MCQ) and interview-based oral examinations were represented in separate arms to emphasise the distinct assessment approaches behind these two evaluation instruments. Additionally, basic YouTube analytics were used to gauge students' level of interest and actual access to video 360 scenarios.

### Ethical considerations

Informed consent was obtained from all individuals who participated in the recorded scenarios. Data collection and analysis were approved by the Ethics Committee of the University (ID 53, received 9 November 2020), and followed the European Union General Data Protection Regulation (GDPR).

### Project participants

The project enrolled 79 students (53 females, 26 males) in the fourth-year of the medical program, who attended the required EM course (hereinafter EMc) in the first semester of the academic year 2020–2021.

Their assessment results at the end of the EMc summative examination were compared to the results of those who had attended the same course, taught by the same faculty during the previous academic year, that is, 2019–2020 (78 students; 61 females and 17 males).

### EM course design and delivery format

Our university employs a lecture-based classical teaching model combined with small-group practical classes. In the EMc, the practical activity was split between the manikin-based simulation centre and ED visits (two-thirds and one-third of the time, respectively). While switching to online classes during the COVID-19 outbreak, the lecture-based model was maintained, although solutions were sought for online teaching and learning to replace the practical sessions.

A team of EM doctors and nurses led by a senior doctor (a faculty member) designed eight teaching scenarios located in a hospital ED. The scripts were prepared based on the existing syllabus of the EM course for fourth-year medical students at our university (three credits), according to international standards [17,18], and covering the patients' assessment, ED management, and treatment of patients with diverse specific pathologies. The full information regarding the learning objectives, target groups, and level of scenario complexity is presented in the web appendix (Supplementary Appendix Table E1a). The scenarios were staged with the ED medical personnel (doctors and nurses from the university-affiliated emergency hospitals) and recorded as video 360 using a GoPro MAX 360 camera, 16.6MP, 5.6K30, Max HyperSmooth, with touch screen and six microphones (https://gopro.com/). The recordings were between 2.13 and 5.44 min long. They were reviewed by two independent experts from the EM department of our university, according to the criteria most frequently used for rating the quality of medical educational videos [19].

**Table 1.** Students' perceptions about the contribution of the video 360 scenarios to their learning, and students' attitudes towards using video 360 for education in emergency medicine and general medical education (anonymous answers).

Variable	Total	Female	Male	*p*-value ^a^
	*n* = 78	*n* = 53	*n* = 25	
Actually prepared	*Questionnaire items Q3–Q10*			
8 items, Cronbach' alpha	0.898	0.846	0.951	NA
Prepared Total40	*Sum of items Q3–Q10*			
Mean ± std.dev.	32.13 ± 5.63	31.49 ± 4.66	33.48 ± 7.2	.073
(min–max)	(12–40)	(20–40)	(12–40)	
Median (IQR)	32 (28–36)	32 (28–35)	35 (28–40)	
Perceived contribution of the 360 scenarios	*Questionnaire items Q11–Q16*			
6 items, Cronbach' alpha	0.919	0.923	0.911	NA
360 scenario Total30	*Sum of items Q11–Q16*			
Mean ± std.dev.	27.35 ± 3.54	27.25 ± 3.61	27.56 ± 3.48	.442
(min–max)	(15–30)	(15–30)	(20–30)	
Median (IQR)	29 (25–30)	29 (25–30)	30 (24–30)	
Knowledge	*Questionnaire item Q17*			
Mean ± std.dev.	4.46 ± 0.73	4.36 ± 0.71	4.68 ± 0.75	.014*
(min–max)	(2–5)	(2–5)	(2–5)	
median (IQR)	5 (4–5)	4 (4–5)	5 (5–5)	
Social competence	*Questionnaire item Q18*			
Mean ± std.dev.	4.17 ± 1.07	4.08 ± 1.09	4.36 ± 1.04	.189
(min–max)	(1–5)	(1–5)	(1–5)	
Median (IQR)	4.5 (4–5)	4 (4–5)	5 (4–5)	
Practical ability	*Questionnaire item Q19*			
Mean ± std.dev.	4.15 ± 1.1	4.11 ± 1.09	4.24 ± 1.17	.447
(min–max)	(1–5)	(1–5)	(1–5)	
Median (IQR)	5 (4–5)	4 (3–5)	5 (4–5)	
360 scenario interest	*Questionnaire item Q20*			
Mean ± std.dev.	4.74 ± 0.49	4.75 ± 0.48	4.72 ± 0.54	.861
(min–max)	(3–5)	(3–5)	(3–5)	
Median (IQR)	5 (5–5)	5 (5–5)	5 (5–5)	
360 scenario extent	*Questionnaire item Q21*			
Mean ± std.dev.	4.74 ± 0.32	4.75 ± 0.59	4.72 ± 0.54	.543
(min–max)	(3–5)	(3–5)	(3–5)	
Median (IQR)	5 (5–5)	5 (5–5)	5 (5–5)	
My ICT ability	*Questionnaire item Q22*			
Mean ± std.dev.	3.94 ± 0.92	3.77 ± 0.91	4.28 ± 0.84	.024*
(min–max)	(2–5)	(2–5)	(3–5)	
Median (IQR)	4 (3–5)	4 (3–4)	5 (4–5)	

^a^ Mann–Whitney test (non-parametric).

Statistical significance: * *p* < .05.

Abbreviations: IQR: Inter-Quartile Range; ICT: Information and Communication Technology; NA: Not Applicable.

All videos were uploaded on YouTube as unlisted videos and made available to students. YouTube hosting services were chosen based on a number of advantages over other alternatives: cross-platform availability, a wide range of supported input file formats, and high popularity—thus implying students' familiarity, free services, and basic analytical tools.

Each scenario was introduced to the students during an online practical session after a theoretical background review and a specific study-case presentation. The large class was split into small groups of five to eight students to discuss the ED assessment and case management. During the discussions, the groups were visited by a moderating teacher who employed the SimMon App (https://simmon-app.com/) to simulate the patient’s monitor. The video recording was available to the students to explore either synchronously or asynchronously (e.g. for further independent study). Similar to the previous year's in-person sessions, a debriefing with all the students followed, emphasising the teaching points.

### Measurement tools and data collection

#### Students' perceptions and attitudes

We measured students' perceptions about the contribution of the video 360 scenarios to their learning, and their attitudes towards the use of video 360 for education in EM and in general medical education using a 23-item online questionnaire (implemented using Google Forms, with anonymous answers). The Likert-type scale questions probed: (a) students' perceived preparedness for EM practical challenges (eight items); (b) the perceived contribution of the video 360 scenarios to EM learning (six items); (c) students' perceived levels of knowledge, social competence, and practical ability in EM (one item for each); (d) students' interest in the video 360 technology (one item) and its use in medical education (one item); and (e) each person's subjective ability to use information and communication technology (ICT, one item). The full questionnaire is presented in the web appendix (Supplementary Appendix Table E2).

**Table 2.** Results of students' assessment in January 2021 (video 360 scenarios employed) and January 2020 (on-site teaching, including manikin simulations; no video scenarios).

Variable	Total	Female	Male	*p*-value^a^
2021: AssessTotal45	*n* = 79	*n* = 53	*n* = 26	
Mean ± std.dev.	39.371 ± 8.07	40.31 ± 7.4	39.31 ± 8.06	.804
(min–max)	(9–45)	(11–45)	(9–45)	
Median (IQR)	42 (40–45)	43 (41–44)	42 (37–45)	
2021: Grade for oral/practical assessment	*n* = 79	*n* = 53	*n* = 26	
Mean ± std.dev.	9.53 ± 1.15	9.73 ± 0.918	9.27 ± 1.34	.099
(min–max)	(5–10)	(5–10)	(5–10)	
Median (IQR)	10 (10–10)	10 (10–10)	10 (9–10)	
2021: MCQ (%)	*n* = 77^b^	*n* = 51^b^	*n* = 26	
Mean ± std.dev.	92.52 ± 4.57	92.31 ± 4.58	92.96 ± 4.61	.548
(min–max)	(79–99)	(80–99)	(79–99)	
Median (IQR)	94 (91–95)	93 (89.5–95.5)	94 (92–95)	
2021: Correlation between AssessTotal45 and Grade for oral/practical assessment: *R* = 0.665 (*p* < .001)				
2021: Correlation between MCQ and Grade for oral/practical assessment: *R* = 0.279 (*p* = .014)				
2020: Grade for oral/practical assessment	*n* = 78	*n* = 61	*n* = 17	
Mean ± std.dev.	8.7 ± 0.98	8.7 ± 0.95	8.71 ± 1.1	.682
(min–max)	(6–10)	(6–10)	(6–10)	
Median (IQR)	9 (8–9)	9 (8–9)	9 (9–9)	
2020: MCQ (%)	*n* = 78	*n* = 61	*n* = 17	
Mean ± std.dev.	76.67 ± 18.77	78 ± 17.96	71.76 ± 21.28	.271
(min–max)	(40–100)	(40–100)	(40–100)	
Median (IQR)	80 (60–90)	80 (60–90)	70 (60–90)	
2020: Correlation between MCQ and Grade for oral/practical assessment: *R* = 0.534 (*p* < .001)				

Grade-related assessments were similar in the two examination sessions, with a supplementary assessment in 2021, based on the nine explicit 5-point criteria (AssessTotal45). ^a^Mann–Whitney test (non-parametric).

^b^Two female students sat the MCQ examination in a formal setting different from their colleagues and were not included in this statistic.

Abbreviations: IQR: Inter-Quartile Range; MCQ: Multiple Choice Questionnaire; R: Spearman coefficient of correlation (non-parametric).

The questionnaire was specifically designed for this project (by faculty members) and no prior formal validation was conducted. The face validity of the questionnaire (namely its relevance to EMc students and this particular project) was assured by employing a Delphi technique in the development process. The reliability of responses for the specific sample of participants in this project was assessed by the internal consistency estimated using the Cronbach's alpha coefficient for each of the two subscales regarding: (a) students' perceived preparedness for EM practical challenges; and (b) the perceived contribution of the video 360 scenarios to EM learning.

#### Academic performance assessment

We assessed students' performance in three independent evaluations: (a) an interview-based oral examination using a novel case scenario (similar to the recorded scenarios) and the SimMon App used for indicating the changes in the patient's medical condition; this examination employed eight 5-point scales incorporating specific EM requirements (assessment of the patient, ability to formulate the diagnosis, ability to formulate thorough differential diagnosis, planning appropriate investigations, prescribing the treatment, description of procedure, ECG interpretation, correctitude in following the cardio-pulmonary resuscitation [CPR] algorithm) and a supplementary 5-point scale called the "Integrated Thought Process", aimed at grasping the expected ability to actually put the theoretical knowledge into practice; (b) a comprehensive assessment in parallel with (a), conducted by an independent evaluator, and following the same examination procedure as during the previous academic year; this was summarised as an overall grade (between 1 and 10); and (c) a MCQ, taken as an online examination; it also followed the same procedure and used the same question-bank as during the previous academic year.

The rubric for the nine criteria employed in the (a) interview-based oral examination was developed by a panel of EM faculty members, hospitalists, and nurses. The rubric for (b) comprehensive oral assessment was developed and applied during previous academic years. The interviews were conducted by a faculty member who graded the comprehensive examination.

The MCQ repository was constructed over the last seven years by a panel of faculty members following the Delphi technique. The initial core consisted of 200 MCQ items with a range of difficulty/complexity from 1 to 5 (the higher the value, the higher the complexity). After each examination, items with extremely low or extremely high response rates were discarded. Presently, the repository consists of 250 questions for each of the eight chapters in the course syllabus, normally distributed over the full range of difficulty/complexity. In required courses for undergraduate students (such as the present educational project), the testing questionnaires consist of 50 questions on levels 2–3, which are uniformly randomised from the repository.

#### Video progress and performance analytics

We employed YouTube analytics as a complement to questionnaire's measurements regarding EMc students' interest in video scenarios, and their level of engagement. For each scenario, basic key metrics were used: the number of views, the average viewing duration, and the average viewing percentage. Supplementary Appendix Table E1a in the web appendix shows this information as an intrinsic part of the scenarios' characteristics, for they illustrate both the viewer interest and retention.

We assumed the access metrics reflect on students' interest for the video 360 scenarios as EMc learning instruments. No subscription or identification was required for accessing the video scenarios; therefore these data were treated as aggregate information.

### Data analysis

In all assessments and questionnaires in this study, the collected data consisted of scores and ranks; they followed non-normal distributions. Shapiro–Wilk statistical test was applied for testing the normality. For all numerical variables, descriptive statistics comprised the range (min‒max), mean, standard deviation, median, and inter-quartile range.

#### Questionnaires' data

For the two subscales of actual preparedness for the EM challenges and perceived contribution of the video recordings to the learning (parts of the anonymous questionnaires), the actual reliability of measurements was assessed based on Cronbach's alpha. Values greater than 0.8 were considered to indicate good internal consistency.

Scorings' descriptive statistics were determined for the total sample of EMc students and on separate groups of female and male participants. Mann–Whitney non-parametric statistical test was applied to compare distributions across the two gender groups. Spearman coefficient of correlation was used to quantify the strength and significance of the relationship between different perceptions and attitudes across the sample of EMc students.

#### Assessment data

On all three assessment arms, descriptive statistics were determined for the total sample of EMc students, and on separate groups of female and male participants. Mann-Whitney non-parametric statistical test was applied to compare distributions across the two gender groups. Spearman coefficient of correlation was used to determine the strength and significance of the relationship between: (a) oral and MCQ examinations; and (b) the two independent oral examinations. The correlation analysis was irrespective of participants' gender. Spearman correlation was also used to investigate the relationship between different assessment criteria employed in the interview-based oral examination.

#### YouTube analytics

Descriptive statistics were determined on aggregate data regarding the access of video 360 scenarios. No gender information was collected or analysed.

Statistical analysis was performed at a 95% level of confidence (i.e. 5% level of statistical significance). All reported probability values were two-tailed. Data analysis was performed using the IBM SPSS trial version and R 3.6.3.

## Results

All 79 students enrolled in the required EM course during the first semester of the academic year 2020–2021 took both the examinations (MCQ and interview-based) and 78 of them (53 females and 25 males) completed the anonymous questionnaire. As students in the fourth year of the medical program, their age range was 22–23 years.

### Students' perceptions and attitudes towards video 360 in medical education

The results regarding students' perceptions of the contribution of the video 360 scenarios to their learning, and their attitudes towards the use of video 360 for education are presented in Table 1. The subscales of actual preparedness and perceived contribution of the video 360 scenarios to students' learning proved good reliability (high values of Cronbach's alpha coefficients). The total scores for each subscale were high (total score in the upper 20 percent), irrespective of students' gender. Female medical students were significantly less confident about their ICT abilities and acquired EM knowledge. All students regarded the video 360 scenarios as highly interesting, useful, and worthy of use on a wider scale in medical education. Supplementary Appendix Table E3 in the web appendix presents the matrix of the coefficients of correlation for students' perceptions and attitudes.

**Table 3.** Matrix of the coefficients of correlation for the nine explicit five-point criteria employed in the independent assessment during the interview-based oral/practical evaluation.

*N* = 79 students in total		Patient evaluation	Integrated thought process	Diagnosis	Differential diagnosis	Investigation plan	Treatment	CPR algorithm	Descriptive procedure	ECG interpretation
Patient evaluation	*R*	1.000	.458**	.501**	.510**	.525**	.450**	.471**	.231*	.230*
	*p*	.	<.001	<.001	<.001	<.001	<.001	<.001	.041	.042
Integrated thought process	*R*	.458**	1.000	.407**	.375**	.453**	.523**	.476**	.541**	.529**
	*p*	<.001	.	<.001	.001	<.001	<.001	<.001	<.001	<.001
Diagnosis	*R*	.501**	.407**	1.000	.877**	.751**	.568**	.536**	.395**	.506**
	*p*	<.001	<.001	.	<.001	<.001	<.001	<.001	<.001	<.001
Differential diagnosis	*R*	.510**	.375**	.877**	1.000	.864**	.590**	.531**	.403**	.522**
	*p*	<.001	.001	<.001	.	<.001	<.001	<.001	<.001	<.001
Investigation plan	*R*	.525**	.453**	.751**	.864**	1.000	.668**	.591**	.406**	.499**
	*p*	<.001	<.001	<.001	<.001	.	<.001	<.001	<.001	<.001
Treatment	*R*	.450**	.523**	.568**	.590**	.668**	1.000	.736**	.484**	.555**
	*p*	<.001	<.001	<.001	<.001	<.001	.	<.001	<.001	<.001
CPR algorithm	*R*	.471**	.476**	.536**	.531**	.591**	.736**	1.000	.424**	.572**
	*p*	<.001	<.001	<.001	<.001	<.001	<.001	.	<.001	<.001
Descriptive procedures	*R*	.231*	.541**	.395**	.403**	.406**	.484**	.424**	1.000	.697**
	*p*	.041	<.001	<.001	<.001	<.001	<.001	<.001	.	<.001
ECG interpretation	*R*	.230*	.529**	.506**	.522**	.499**	.555**	.572**	.697**	1.000
	*p*	.042	<.001	<.001	<.001	<.001	<.001	<.001	<.001	.

Statistically significant values of coefficients *R* over 0.6 were marked in bold.

Statistical significance: * *p* < .05; ** *p* < .01.

Abbreviations: CPR: Cardio-Pulmonary Resuscitation; ECG: electrocardiogram; p: statistical significance; R: Spearman coefficient of correlation (non-parametric).

### Students' academic assessment in January 2021

Table 2 shows the results of students' assessments in January 2021 and January 2020. There was a strong and statistically significant correlation between the two independent interview-based oral examinations in 2021. The grades for oral/practical assessment were higher in January 2021 than in January 2020. The scores in the MCQ examination were ∼20% higher in January 2021 compared to those in the previous year. The corresponding standard deviation was considerably smaller in 2021, indicating less variability in MCQ scores. No gender differences were observed in any of the assessments (Table 2).

Table 3 presents the matrix of the coefficients of correlation for the nine explicit 5-point criteria employed in the independent assessment during the interview-based oral/practical evaluation, developed for the online examination in January 2021. It is noteworthy that both "Patient Evaluation" and "Integrated Thought Process" were weakly correlated to any other assessment criteria (although their correlation was statistically significant). Each of the other seven criteria strongly correlated with at least one criterion.

### Video progress and performance analytics

In order to corroborate the anonymously declared perceptions and attitudes with objective data, the basic analytical instruments offered by YouTube were used to evaluate the actual access of video 360 scenarios on an individual basis. The recordings had between 175 and 411 views, with an average viewing percentage of 57.93 ± 6.53. The full access data and statistics are presented in the web appendix (Supplementary Appendix Table E1b).

## Discussion

The anonymous online questionnaires measured the medical students' high interest, perceived usefulness, and acceptance of the video 360 technology, with scorings in the upper 20%. Academic assessment at the end of EMc in January 2021 proved good performance in both summative MCQ and interview-based oral/practical evaluations, with a significant correlation between them, and better results compared to similar assessments in January 2020. Statistics on aggregate data collected by YouTube analytics confirmed the students' expressed interest in the scenarios.

### Students' perceptions and attitudes towards Video 360

Students' perceived usefulness of the video 360 technology was high, with almost unanimous high interest in this technology and the desire to employ it in other medical courses. Their declared interest was corroborated by their independent access to the unlisted video clips on YouTube. Although aggregate information, these statistics suggest that students independently accessed the video scenarios multiple times.

The results of our pilot educational study confirmed other reported perceptions regarding the use of VR and computer simulations in EM training [11–13,15,20]. In a 2021 study by Chan et al. [21], the video 360 technology was explicitly compared with traditional video recordings as regards their respective abilities to promote student engagement in a clinical anatomy and imaging integrative laboratory experience. At each time-point, the average self-reported engagement in the video 360 group was higher, with statistically significant differences in terms of learning stimulation, enjoyment, practicality, and interest in the technology itself.

Compared to other studies, an important contribution of our project to the literature is that student's participation in the educational activities employing video 360 scenarios was not voluntary; all participants in the required EM course were enrolled and finally sat the required summative assessment at the end of the course. The majority (78 out of 79 participants) also provided anonymous feedback and valued the video 360 technology as contributing substantially to their knowledge and preparedness, with high scores towards extending this approach in medical education. Since this was a required course, the feedback would be less affected by certain bias, such as courtesy bias or demand characteristics.

### Effectiveness of video 360 scenarios

The video 360 scenarios in our project tried to mimic the on-site training sessions as a substitute for the manikin training or the real patient case scenario. Our findings support the effectiveness of employing the video 360 technology in teaching EM. The results of the 2021 assessment were generally better than those of 2020 (when on-site teaching was employed): approximately one grading point higher for the oral examination and about 20% higher for the MCQ examination, with less variability in both. Although female medical students seemed less confident about their ICT abilities and their acquired EM knowledge, the actual examination results proved that there were no such differences. The strong correlation between the results of the two independent and parallel oral examinations supports the validity of the assessment results; thus, the educational effectiveness of video 360 scenarios in EM.

Video-based teaching has been a successful part of medical education since the beginning of the 2010s [22–24]. In fact, the video 360 technology has been hailed as a new tool that offers a unique sense of presence and immersion that cannot be achieved with traditional videos [10]. In a single-blinded randomised cross-over study with 40 students, Harrington et al. [20] compared video 360 recordings with traditional video recordings for teaching surgical procedures and found no significant difference in information retention as measured in an MCQ test about the video content—although the reported levels of engagement and attentiveness were higher for video 360 recordings. In contrast to enthusiastic engagement, mixed results regarding the actual impact on learning were reported by most studies conducted before 2019 [25,26]. All participants had voluntary enrolment and there were few participants in the experimental video groups.

In their recent literature review including 64 articles, Pirker and Dengel [16] focussed on identifying the educational potential of the video 360 technology, and more than a quarter (28.1%) of the selected articles addressed topics from the medicine or healthcare fields. Positive effects on learning were reported in terms of improved knowledge retention, increased motivation, and enhanced performance. Regarding human factors, most of the selected papers reported positive effects on presence, perception, engagement, emotions, and empathy. Increased cognitive load and negative affectivity were among the only few disadvantages reported [16].

More recently, Arents et al. [14] reported that video 360 VR was a good option for preparing students to attend new real-life situations, although they did not identify any instance of improvement over the long term in the case of either general or specific knowledge on gentle caesarean sections after students' required internship. The cohort of 89 medical students, although split into a video 360 group and a conventional control group, was of a similar size to the cohort in our study.

Nonetheless, we are aware that online learning can never fully replace hands-on teaching, and that further on-site training is required for the correct execution of manoeuvres. Practical skills training and subsequent actual practice are irreplaceable in EM education (e.g. airway management techniques; CPR; airway, breathing, circulation, disability, and exposure (ABCDE) evaluation of emergency patients with life-saving manoeuvres involved, etc.).

The independent interview-based assessment according to the nine 5-point criteria—a rubric developed for this project—provided supplementary insight into students' understanding of the EM-specific standards of care and challenges. The weak correlation of patient evaluation and integrated thought process with any other criterion suggests these two criteria as providing comprehensive evaluation not inferred from other abilities or knowledge. The findings on these nine assessment criteria are unique to the present study and would help in designing new scenarios to better address specific EM learning objectives. Furthermore, this insight could be employed in developing educational resources for a wider range of medical specialties.

Another important novel contribution of our research regarding the video 360 technology in medical education consists of a comprehensive analysis of eight different scenarios from multiple EM areas, in connection with other branches of medicine. Each of the previous reports on the video 360 educational impact [14,20,21] used only one video on a single subject or procedure. Our project's scenarios covered resuscitation, assessment of the critical patient, cardiac and respiratory emergencies, trauma, shock, and intoxication.

Summing up, an ED is never quiet, or big enough. Therefore, EM education requires spontaneous and flexible teaching styles, covering a wide range of methods: from formal classes and manikin simulation centres to bedside instruction. The unexpected COVID-19 pandemic created new and, too often, overwhelming challenges in teaching EM. In the face of a highly contagious pandemic, students may unknowingly transmit the virus or contract the disease themselves. The lack of personal protective equipment (PPE) kits prevented students from receiving bedside learning, which had been the norm before the pandemic. Consequently, EM teachers had to devise a compromise between education quality and a safe learning environment [11–15,27,28]. Similar concerns were raised by other medical teachers, especially those directly related to emergency surgery [29–31]. Additionally, the range of pathologies, treatments, and interventions to which students were exposed diminished for all branches of medicine because of the altered patient admittance protocols and treatment algorithms in teaching hospitals, and as per the recommendations of international medical bodies. Even in surgical wards, only life-threatening emergencies were treated, with the procedures and surgical techniques drastically revised, switching to conservative approaches, whenever possible [32–35]. Within and beyond the actual context, video 360 technology is proposed as a viable solution in EM teaching.

### Limitations

This pilot project had inherent limitations:

1. Due to its rather small scale and lack of baseline evaluation, the effects of the educational intervention on the learning outcomes could not be reliably quantified. Therefore, we provided only descriptive statistics for the results of students' assessments.
2. As a pilot endeavour, it was subject to unavoidable beginners' staging flaws; experience accrual and future professional guidance would improve such educational approaches.
3. A certain ascertainment bias was generated because of the participants' enthusiastic involvement and eagerness to surmount the pandemic difficulties (students and teachers, alike). This could be a confounder for both the students' perceptions and attitudes, and the learning effort for the required EM course-end examination.
4. When comparing the assessment results across the two examination sessions in different academic years, multiple supplementary confounders should be honestly acknowledged; that is, not only students' motivation but also different general academic achievements in the two groups of students. Therefore, we opted to provide only descriptive statistics for these measurements, rather than confidence intervals or p-values from statistical testing.

Furthermore, video 360 scenarios were recorded by the ED medical personnel (doctors and nurses from the university-affiliated emergency hospitals), with no professional guidance or previous acting training. Further revision by the development team with debriefing after this pilot project, and peer revision by independent evaluators will certainly add value to such educational scenarios before the next development stage (which would include decision trees for alternative medical outcomes, based on students' decisions at certain critical points). However, the project was designed as a proof of concept; this report is, therefore, intended as a promoter of the teaching approach, rather than the scenarios themselves (which, at this stage, are in Romanian only).

## Conclusion

During the COVID-19 pandemic, medical teachers had to devise innovative methods to ensure the continuity of the education process in a safe and reliable manner. The lessons thus learned should be employed in regular teaching. The video 360 technology in EM education would be a viable solution for hybrid teaching in both large and small classes while using diverse teaching models and curriculum designs. Moreover, it would allow the natural implementation of team-learning approaches, particularly relevant to the EM practice.

In conclusion, employing this accessible and inexpensive educational approach would add value to on-site training by enriching the exposure to a specific ED environment without causing overcrowding, both in the short- and long term. Moreover, the lessons can be directly extended to other medical specialties. Such recorded scenarios would offer the opportunity to asynchronously observe specific environmental details according to trainees' own pace and needs, tailoring their learning experience before actually stepping into a real medical ward.

## Supplementary Material

Supplemental Material

## Data Availability

Additional data are available upon reasonable request.
