# Supplementary material for: Using 360-degree video for teaching emergency medicine during and beyond the COVID-19 pandemic
Source: Ann Med. 2021 Oct 6;53(1):1520–30. doi: 10.1080/07853890.2021.1970219 (PMC8510619; doi:10.1080/07853890.2021.1970219)
Supplement: Supplemental Material [file IANN_A_1970219_SM1718.docx]

**Table E1a.** Characteristics of the 360 scenarios for EM education: 24 March 2021 (loaded on 18th December 2020 and 1st January 2021)

| **Scenario** | **Learning objectives** | **Target group** | **Complexity level (1 to 5)** | **Videoclip duration** | **Views** | **Average viewing time** | **Average viewing %** |
| --- | --- | --- | --- | --- | --- | --- | --- |
| **S1**  Cocaine intoxication | 1. Patient assessment  2. Sympatho-mimetic toxidrome  3. ED management of the patient with cocaine intoxication  3. Managing the team (leadership skills)  4. Teamwork skills (nurse – doctor) | resident medical doctors  nurses  medical students  nursing students | 3 | 4.14 minutes | 175 | 2.22 minutes | 56.3% |
| https://youtu.be/M1SNA06FroE | | | | | | |  |
| **S2**  Car accident - pedestrian hit by a car | 1. Primary survey of polytraumatized patient  2. ED management of tension pneumothorax  3. Teamwork skills (nurse – doctor) | resident medical doctors  nurses  medical students  nursing students | 3 | 3.50 minutes | 411 | 2.06 minutes | 54.8% |
| https://youtu.be/ttD6q7E6PrA | | | | | | | |
| **S3**  Anaphylactic shock | 1. ABCDE assessment  2. ED management of the patient with anaphylactic shock | resident medical doctors  nurses  medical students  nursing students | 3 | 3.29 minutes | 188 | 1.42 minutes | 49% |
| https://youtu.be/nkmAiQEgRo4 | | | | | | | |
| **S4**  Acute myocardial infarction | 1. Patient assessment  2. ED management of the patient with STEMI  3. Management of tachyarrhythmias – VT with pulse  4. Teamwork skills (nurse – doctor) | resident medical doctors  nurses  medical students  nursing students | 4 | 5.44 minutes | 269 | 3.11 minutes | 55.7% |
| https://youtu.be/sGre4fCnyoc | | | | | | | |
| **S5**  Pulmonary edema | 1. Patient assessment  2. Management of the patient with pulmonary edema  3. Non-invasive ventilation for pulmonary edema  4. Teamwork skills (nurse – doctor) | resident medical doctors  nurses  medical students  nursing students | 3 | 5.02 minutes | 246 | 3.08 minutes | 62.4% |
| https://youtu.be/WQhypbRUF4A | | | | | | | |
| **S6**  Pulmonary embolism | 1. Patient assessment  2. Management of the patient with PE  3. Cardiac arrest - non shockable rhythms (PEA)  4. Teamwork skills (nurse – doctor) | resident medical doctors  nurses  medical students  nursing students | 4 | 4.32 minutes | 256 | 2.22 minutes | 53.3% |
| https://youtu.be/-YtlMxofkag | | | | | | | |
| **S7**  ALS non shockable rhythms | 1. Resuscitation algorithm  2. Teamwork skills (nurse-doctor)  3. Leading the resuscitation team | resident medical doctors  nurses  medical students  nursing students | 2 | 2.44 minutes | 217 | 1.41 minutes | 62% |
| https://youtu.be/m1ooVF8umQA | | | | | | | |
| **S8**  ALS shockable rhythms | 1. Resuscitation algorithm  2. Teamwork skills (nurse – doctor)  3. Leading the resuscitation team | resident medical doctors  nurses  medical students  nursing students | 2 | 2.13 minutes | 198 | 1.32 minutes | 69.9% |
| https://youtu.be/Gf4n2xFKhS0 | | | | | | | |
| Abbreviations: ALS, Advanced Life Support; ED, Emergency Department; PEA, Pulseless Electrical Activity; VT, Ventricular Tachycardia | | | | | | | |

**Table E1b.** Viewing statistics for the 360 scenarios for EM education: 24 March 2021 (loaded on 18th December 2020 and 1st January 2021)

| **Scenarios' statistics** (N=8 scenarios) | **mean ± std.dev.** | **min ‒ max** |
| --- | --- | --- |
| Videoclip duration [minutes] | 3.79 ± 1.17 | 2.13 ‒ 5.44 |
| Views | 245 ± 75.02 | 175 ‒ 411 |
| Average viewing time [minutes] | 2.11 ± 0.71 | 1.32 ‒ 3.11 |
| Average viewing % | 57.93 ± 6.53 | 49 ‒ 69.9 |

**Table E2.** Questionnaire used for collecting students' perceptions about the contribution of the video 360 scenarios to their learning, and students' attitudes toward using video 360 for education in emergency medicine and in general medical education. The column *Variable* shows the variable generated from the answers and used in data analysis for assessing students' perceptions and attitudes towards the video 360 technology.

| **No** | **QUESTION** | **Answer options** | **Variable** |
| --- | --- | --- | --- |
| Q1 | I confirm that I read and I understood the above information. I agree to give feedback on the practical emergency medicine (EM) classes. | Yes (required to continue) | ‒ |
| Q2 | Before the COVID-19 pandemic, have you ever participated to a first aid/emergency medicine/resuscitation course? | Yes/No | ‒ |
|  | **After I attended the practical EM classes, I feel confident to …** |  | prepared Total40 |
| Q3 | …recognize a critical patient. | 1 ‒ 5 |  |
| Q4 | …perform a cardio-pulmonary resuscitation. | 1 ‒ 5 |  |
| Q5 | …assess a patient presenting to the Emergency department (ED). | 1 ‒ 5 |  |
| Q6 | …elaborate a treatment plan for a patient presenting to the ED. | 1 ‒ 5 |  |
| Q7 | ...make the differential diagnosis for the most frequent complaints in the ED (chest pain, dyspnea, headache, ..). | 1 ‒ 5 |  |
| Q8 | …perform advance management of the airway (e.g. endotracheal intubation). | 1 ‒ 5 |  |
| Q9 | …perform the primary survey for a multiple traumatized patient. | 1 ‒ 5 |  |
| Q10 | …recognize on the ECG life threatening rhythms. | 1 ‒ 5 |  |
|  | **To these practical EM classes, the 360 video scenarios…** |  | 360 scenario Total30 |
| Q11 | …contributed to the development of clinical abilities. | 1 ‒ 5 |  |
| Q12 | …contributed to the strengthening of the theoretical knowledge. | 1 ‒ 5 |  |
| Q13 | …reproduced the ED environment. | 1 ‒ 5 |  |
| Q14 | …were helping in defining roles in the team. | 1 ‒ 5 |  |
| Q15 | …made the online classes more attractive. | 1 ‒ 5 |  |
| Q16 | …fitted my learning approach. | 1 ‒ 5 |  |
|  | **In conclusion:** |  |  |
| Q17 | The online practical EM classes succeeded to transmit the knowledge needed for the management of a critical patient. | 1 ‒ 5 | knowledge |
| Q18 | The online practical EM classes contributed to the development of my social skills. | 1 ‒ 5 | social Competence |
| Q19 | The online practical EM classes contributed to the development of my clinical skills. | 1 ‒ 5 | practical Ability |
| Q20 | Video 360 is an interesting technology. | 1 ‒ 5 | 360 scenario Interest |
| Q21 | I would like video 360 to be integrated in teaching other medical subjects. | 1 ‒ 5 | 360 scenario Extent |
| Q22 | How would you describe your computer skills? | 1 ‒ 5 | my ICT ability |
| Q23 | Your gender is: | F, M, Other | Gender |

**Table E3.** Matrix of the coefficients of correlation for the students' perceptions and attitudes toward using video 360 scenarios for education in emergency medicine and in general medical education (anonymous answers to the questionnaire in Annex 2). Statistically significant values of coefficients R over 0.6 were marked in bold.

| N=78 answers in total |  | prepared Total40 | 360 scenario Total30 | knowledge | social Competence | practical Ability | 360 scenario Interest | 360 scenario Extent | my ICT ability |
| --- | --- | --- | --- | --- | --- | --- | --- | --- | --- |
| prepared Total40 | R | 1.000 | **.659**^**^ | .569^**^ | **.685**^**^ | **.689**^**^ | .461^**^ | .326^**^ | .353^**^ |
|  | p | . | <0.001 | <0.001 | <0.001 | <0.001 | <0.001 | 0.004 | 0.002 |
| 360 scenario Total30 | R | .659^**^ | 1.000 | .515^**^ | **.663**^**^ | **.671**^**^ | .579^**^ | .481^**^ | .325^**^ |
|  | p | <0.001 | . | <0.001 | <0.001 | <0.001 | <0.001 | <0.001 | 0.0004 |
| knowledge | R | .569^**^ | .515^**^ | 1.000 | **.743**^**^ | **.631**^**^ | .328^**^ | .200 | .255^*^ |
|  | p | <0.001 | <0.001 | . | <0.001 | <0.001 | 0.003 | 0.078 | 0.024 |
| social Competence | R | .685^**^ | .663^**^ | .743^**^ | 1.000 | **.821**^**^ | .517^**^ | .339^**^ | .356^**^ |
|  | p | <0.001 | <0.001 | <0.001 | . | <0.001 | <0.001 | 0.002 | 0.001 |
| practical Ability | R | .689^**^ | **.671**^**^ | **.631**^**^ | **.821**^**^ | 1.000 | .486^**^ | .332^**^ | .317^**^ |
|  | p | <0.001 | <0.001 | <0.001 | <0.001 | . | <0.001 | 0.003 | 0.005 |
| 360 scenario Interest | R | .461^**^ | .579^**^ | .328^**^ | .517^**^ | .486^**^ | 1.000 | **.667**^**^ | .233^*^ |
|  | p | <0.001 | <0.001 | 0.003 | <0.001 | <0.001 | . | <0.001 | 0.040 |
| 360 scenario Extent | R | .326^**^ | .481^**^ | .200 | .339^**^ | .332^**^ | **.667**^**^ | 1.000 | .326^**^ |
|  | p | 0.004 | <0.001 | 0.078 | 0.002 | 0.003 | <0.001 | . | 0.004 |
| my ICT ability | R | .353^**^ | .325^**^ | .255^*^ | .356^**^ | .317^**^ | .233^*^ | .326^**^ | 1.000 |
|  | p | 0.002 | 0.004 | 0.024 | 0.001 | 0.005 | 0.040 | 0.004 | . |
| Statistical significance: * p < 0.05; ** p < 0.01 | | | | | | | | | |
| Abbreviations: ICT, Information and Communication Technology; p, statistical significance; R, Spearman coefficient of correlation (nonparametric) | | | | | | | | | |
